# Apolipoprotein E knockout, but not cholesteryl ester transfer protein (CETP)-associated high-density lipoprotein cholesterol (HDL-C) lowering, exacerbates muscle wasting in dysferlin-null mice

**DOI:** 10.1186/s12944-024-02227-5

**Published:** 2024-08-13

**Authors:** Zeren Sun, Zoe White, Marine Theret, Pascal Bernatchez

**Affiliations:** 1 Department of Anesthesiology, Pharmacology & Therapeutics, University of British Columbia (UBC), 217-2176 Health Sciences Mall, Vancouver, BC V6T 1Z3 Canada; 2 UBC Centre for Heart Lung Innovation, St. Paul’s Hospital, Vancouver, BC Canada; 3 School of Biomedical Engineering, Department of Medical Genetics, University of British Columbia (UBC), Vancouver, BC Canada

**Keywords:** Muscular dystrophy, CETP, Dysferlin, HDL-C, Cholesterol

## Abstract

**Background:**

Dysferlin-deficient limb-girdle muscular dystrophy type 2B (Dysf) mice are notorious for their mild phenotype. Raising plasma total cholesterol (CHOL) via apolipoprotein E (ApoE) knockout (KO) drastically exacerbates muscle wasting in Dysf mice. However, dysferlinopathic patients have abnormally reduced plasma high-density lipoprotein cholesterol (HDL-C) levels. The current study aimed to determine whether HDL-C lowering can exacerbate the mild phenotype of dysferlin-null mice.

**Methods:**

Human cholesteryl ester transfer protein (CETP), a plasma lipid transfer protein not found in mice that reduces HDL-C, and/or its optimal adapter protein human apolipoprotein B (ApoB), were overexpressed in Dysf mice. Mice received a 2% cholesterol diet from 2 months of age and characterized through ambulatory and hanging functional tests, plasma analyses, and muscle histology.

**Results:**

CETP/ApoB expression in Dysf mice caused reduced HDL-C (54.5%) and elevated ratio of CHOL/HDL-C (181.3%) compared to control Dysf mice in plasma, but without raising CHOL. Compared to the severe muscle pathology found in high CHOL Dysf/ApoE double knockout mice, Dysf/CETP/ApoB mice did not show significant changes in ambulation, hanging capacity, increases in damaged area, collagen deposition, or decreases in cross-sectional area and healthy myofibre coverage.

**Conclusions:**

CETP/ApoB over-expression in Dysf mice decreases HDL-C without increasing CHOL or exacerbating muscle pathology. High CHOL or nonHDL-C caused by ApoE KO, rather than low HDL-C, likely lead to rodent muscular dystrophy phenotype humanization.

**Supplementary Information:**

The online version contains supplementary material available at 10.1186/s12944-024-02227-5.

## Background

Muscular dystrophies (MDs) are a group of neuromuscular diseases often caused by mutations to genes that encode for proteins critical sarcolemma maintenance and myofibre extracellular matrix connection [1]. Mutations of dysferlin (Dysf), a key sarcolemma integrity protein, lead to dysferlinopathies including limb-girdle muscular dystrophy type 2B (LGMD2B) due to impaired calcium-dependent fusion of lipid patches and transmembrane protein trafficking during sarcolemma resealing after mechanical injury [2–5]. This results in symptomatic appendicular muscle wasting in early adulthood, which eventually leads to complete loss of mobility a decade after diagnosis [6]. To date, no curative treatment for LGMD2B exists.

Previous studies reported that LGMD2B as well as Duchenne, Becker and other types of MDs are correlated to high prevalence of dyslipidemia, manifested by abnormal levels of plasma triglycerides (TG), total cholesterol (CHOL) [7–9], and specifically low high-density lipoprotein cholesterol (HDL-C) in LGMD2B cases, independent of stratification by age or gender [9]. HDL particles are well documented to protect against atherosclerosis and cardiovascular diseases (CVDs) in part due to their ability to promote reverse cholesterol transport (RCT), a process that clears excessive cholesterol from peripheral tissues, exerting antioxidant, anti-inflammatory, and anti-thrombotic benefits [10, 11]. A metabolic state of dyslipidemia appears to be linked to MD-causing mutations rather than secondary to the muscle wasting process, as dyslipidemia can be observed in unaffected heterozygous carriers [8] and free cholesterol accumulation in MD muscles [9] is the likely source of dyslipidemia. Of note, mild rodent models of MD show severe muscle wasting exacerbation by inactivating the apolipoprotein E (ApoE) gene, which is critical to circulating lipoprotein clearance and leads to CHOL and nonHDL-C elevations. Dysferlin/ApoE double knockout (Dysf/ApoE) mice present humanization of muscle phenotypes including fatty/fibrotic infiltration, leading to severe ambulatory dysfunction, a process accelerated by high-fat diet (HFD) [12] and even further by high-cholesterol dietary supplementation [13], providing causal evidence that abnormal cholesterol and lipoprotein metabolism play a detrimental role in MD.

Cholesteryl ester transfer protein (CETP) is plasma lipid transfer protein expressed by humans but absent in mice that mediates the exchange of neutral lipids between lipoproteins, such as TG for cholesterol esters between HDL and other apolipoprotein B (ApoB)-containing lipoproteins [14]. CETP activity results in the net reduction of HDL-C and increases in nonHDL-C [15, 16]. As mice are notorious for their non-humanized lipid profile, especially abundant HDL-C, and that LGMD2B patients have lower HDL-C, the hypothesis of this study was that introducing CETP and its optimal adapter protein human ApoB, a key constituent of nonHDL particles, rather than mouse ApoB, in Dysf mice would reduce HDL-C, increase nonHDL-C and exacerbate MD severity similar to that observed in Dysf/ApoE mice. Empirically, CETP transgene contributes to humanized plasma CETP concentration (∼ 2 ug/ml) and reduced plasma HDL-C levels in mice [17, 18]. On the other hand, others reported that ApoB-100 transgenic mice with dietary high-cholesterol intervention showed elevated serum CHOL and LDL-C levels, without interfering reducing HDL-C levels [19]. As CETP is not expressed in mice and ApoB is found at the surface of atherogenic lipoproteins, the present study was aimed at investigating the role of human CETP/ApoB expression in LGMD2B-related HDL-C lowering and muscle wasting by generating Dysf/CETP/ApoB mice. As patients express ApoE, CETP, and ApoB, this study would also improve LGMD2B modeling and humanization by comparing the phenotype of CETP/ApoB transgenic Dysf mice to that of Dysf mice lacking ApoE.

## Methods

### Animal studies and modeling

Animal procedures were designed and carried out conforming to the guidelines and regulations set by the Animal Care Committee and the Animal Ethics Committee at University of British Columbia (UBC), Vancouver, BC, Canada. Animal housing was maintained in a temperature-controlled facility equipped with ventilation system and a 12/12 hrs light/dark switch. Expression of human CETP and ApoB in Dysf mice was achieved by crossing Dysf^−/−^ mice (B6.129-Dysf^tm1Kcam^; The Jackson Laboratory, Bar Harbor, Maine, USA) with the CETP/ApoB transgenic mouse (CETP^+/−^ApoB^+/−^; Taconic Biosciences, Rensselaer, NY, USA) to generate Dysf^−/−^CETP^+/−^ and Dysf^−/−^ApoB^+/−^ littermates. These littermates were subsequently crossbred to generate Dysf^−/−^ (Dysf), Dysf^−/−^CETP^+/−^ (Dysf/CETP), Dysf^−/−^ApoB^+/−^ (Dysf/ApoB), and Dysf^−/−^CETP^+/−^ApoB^+/−^ (Dysf/CETP/ApoB) experimental mice. Another cohort of Dysf/ApoE double knockout mice was generated as by crossbreeding Dysf^−/−^/ApoE^+/−^ mice [12, 13]. Mice were weaned at 3–4 weeks, supplemented ad libitum and received a modified chow-based diet (LabDiet 5001) with 2% cholesterol supplement (TD.0009393; 13.1% total fat and 3.7% sucrose by weight; TestDiet, Richmond, IN, USA) from 2 months of age until 5 months (for all mice) or 11 months of age (for Dysf with CETP/ApoB transgene only).

### Gait tracking and gross hindlimb morphology

Ambulatory function was assessed by gait tracking. Briefly, hind paws of mice were painted with color ink, and footprints were recorded on a 1.5 m length tracking paper after mice passing through. The step length was measured by the distance between corresponding positions of two sequential footprints on the same side. The average step length for each mouse was calculated from measurements of both feet with triplicated tracking. Photographs of hindlimbs and isolated muscles were captured upon euthanasia and removal of the epidermis, to assess the gross morphology.

### Hanging test

A four-limb hanging test was performed to evaluate general muscle strength, balance, and endurance, adapted from previously described [20]. Briefly, the mouse was placed on a cage grid, 15 cm above a surface with soft bedding. The hanging time was recorded starting from turning the grid upside down (mouse grasped the grid with four limbs) until the mouse fell to the surface. Each mouse was given three consecutive trials, with a 30-sec resting interval between individual trials. Test was terminated after a maximum hanging time of 360 s was achieved in individual trial or after completing 3 trials. Body weight was recorded before the test, and the results were quantified by calculating the Holding Impulse (g*sec) = Body weight (g) × maximum Hanging Time (sec).

### Tissue processing and histology analysis

Mice were anaesthetized (5% v/v isoflurane mixed with O_2_) and then euthanized via cardiac puncture and Krebs buffer perfusion (118 mmol/L NaCl, 22.5 mmol/L NaHCO_3_, 4 mmol/L KCl, 1.2 mmol/L NaH_2_PO4, 2 mmol/L CaCl_2_, 2 mmol/L MgCl_2_ and 11 mmol/L dextrose). Quadriceps (QUA), gastrocnemius (GAS), tibialis anterior (TA), and triceps (TRI) muscles were collected and weighed upon euthanasia. 10% formalin was used for QUA and TRI muscle fixation (48 h) followed by storage in 70% Ethanol. Muscles were cross-sectionally cut and both halves were embedded in paraffin. Embedded muscles were cut into 5 μm thick sections for Masson’s trichrome or hematoxylin and eosin (H&E) staining as previously described [12, 21]. Full-slide scanning was operated by an Aperio digital slide scanner. In Masson’s trichrome staining images, damaged areas were measured by manually tracing non-myofibre areas including inflammation, necrosis, and fatty infiltrations, and results are presented as the proportion of total cross-sectional area (% damage). Healthy myofibre areas (% myofibre) were further calculated by subtracting damaged areas from the total cross-sectional area. Fibrosis was labeled by collagen-positive areas (% collagen) and quantified by a positive pixel counting algorithm set in Aperio ImageScope software (0.66 hue value, 0.25 hue width).

### Plasma lipoprotein analysis

Blood was collected via cardiac puncture after anesthesia and placed in tubes with heparin. Separation of plasma was conducted by spinning down blood at 4,000 RPM for 10 min at 4 °C, and the upper layer was transferred to -80 °C for long-term storage. Plasma lipoproteins (CHOL, HDL-C, TG) were analyzed by the Siemens Advia 1800 system (Providence HealthCare, St Paul’s Hospital, Vancouver, BC, Canada), as previously reported [9, 12, 13]. The nonHDL-C level was calculated by subtracting HDL-C from CHOL.

### Analysis of intramuscular lipid accumulation

Adipocytes were visualized by perilipin immunofluorescent staining on paraffin muscle sections (5 μm thickness) as previously described [13]. Briefly, muscle sections were deparaffinized and heated in citrate buffer (10 mM) at 90 °C for antigen retrieval. Cooled slides were then washed twice with phosphate-buffered saline (PBS) and blocked with a blocking buffer (PBS containing 3% normal goat serum and 0.3% triton X-100) for 60 min at room temperature (RT). Next, muscle sections were incubated overnight in anti-perilipin antibody (#D1D8; 1:100 in blocking buffer; Cell Signaling Technology, Danvers, MA, USA) at 4 °C. Slides were washed with PBS (5 min for 3 times) followed by incubation in goat anti-rabbit Alexa Fluor™ 488 secondary antibody (#A-21070; 1:500 in blocking buffer; Thermo Fisher Scientific, Waltham, MA, USA) for 2 h at RT. After another serial of PBS wash (5 min for 3 times), sections were mounted with Fluoroshield™ mounting medium with DAPI (#F6057; Sigma-Aldrich, Burlington, MA, USA). Slides were scanned with the Nikon Eclipse Ni microscope/camera system to capture full-section images and 10x magnifications. Percentages of adipocytes were quantified using ImageJ.

### Statistical analysis

Statistical analysis and graph production were performed in GraphPad Prism 6. A one-way ANOVA with Tukey’s post-hoc test was used to compare means between three or more groups. Figures show data as mean ± standard deviation (SD). Figure legends contained specification of the number of animals per group. *P* values < 0.05 were defined as statistically significant.

## Results

**Fig. 1:**
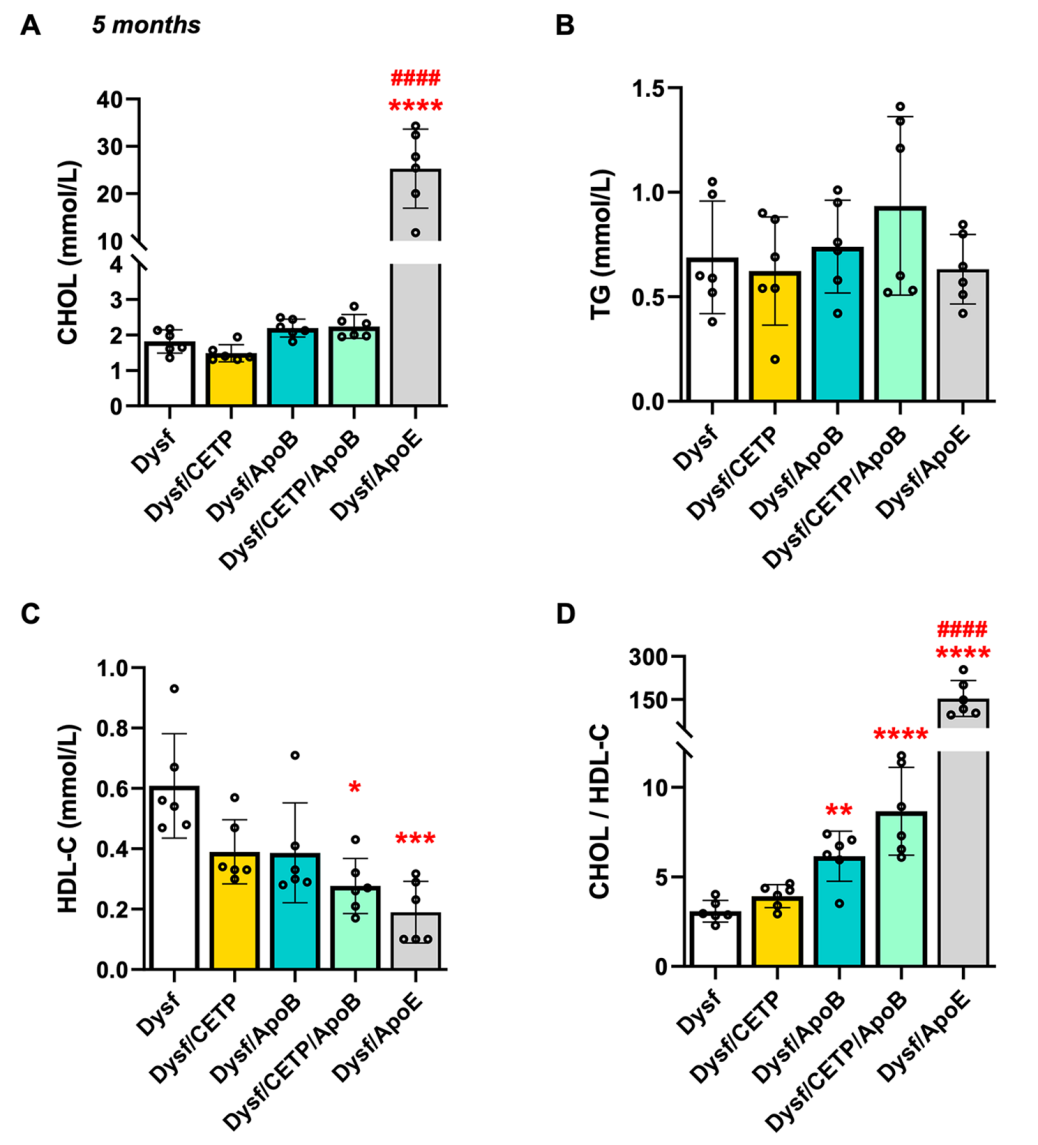
CETP/ApoB transgenes decreased plasma high-density lipoprotein cholesterol (HDL-C) portion in 5-month-old adult Dysf mice. Plasma collected from Dysf, Dysf/CETP, Dysf/ApoB, Dysf/CETP/ApoB, and Dysf/ApoE mice (*n* = 6) was analyzed for (**A**) total cholesterol (CHOL), (**B**) total triglycerides (TG), and (**C**) HDL-cholesterol levels. (**D**) CHOL/HDL-C ratio was calculated. Data were presented as Mean ± SD. *P < 0.05, *P < 0.01, ***P < 0.001, ****P < 0.0001 compared to Dysf; ^####^*P* < 0.0001 compared to Dysf/CETP/ApoB

CETP/ApoB transgenes were expressed in Dysf mice to humanize their plasma lipid profile. As shown in Fig. 1, 5-month-old Dysf mice expressing human CETP, human ApoB, or both human CETP/ApoB transgenes showed similar levels of plasma CHOL and TG, whereas positive control Dysf/ApoE mice showed a 1291.7% increase in plasma CHOL compared to Dysf mice (Fig. 1A-B). However, Dysf/CETP/ApoB mice showed a significant 54.5% decrease in plasma HDL-C compared to Dysf mice, while Dysf/ApoB and Dysf/CETP/ApoB mice showed 99.6% and 181.3% increases in CHOL/HDL-C ratio, respectively, a key risk factor for CVDs (Fig. 1C-D). These results indicated that human CETP/ApoB transgene combined with a high cholesterol diet lower plasma HDL-C levels in adult Dysf mice, while only Dysf/ApoE showed severe hypercholesterolemia with high nonHDL-C levels.

**Fig. 2:**
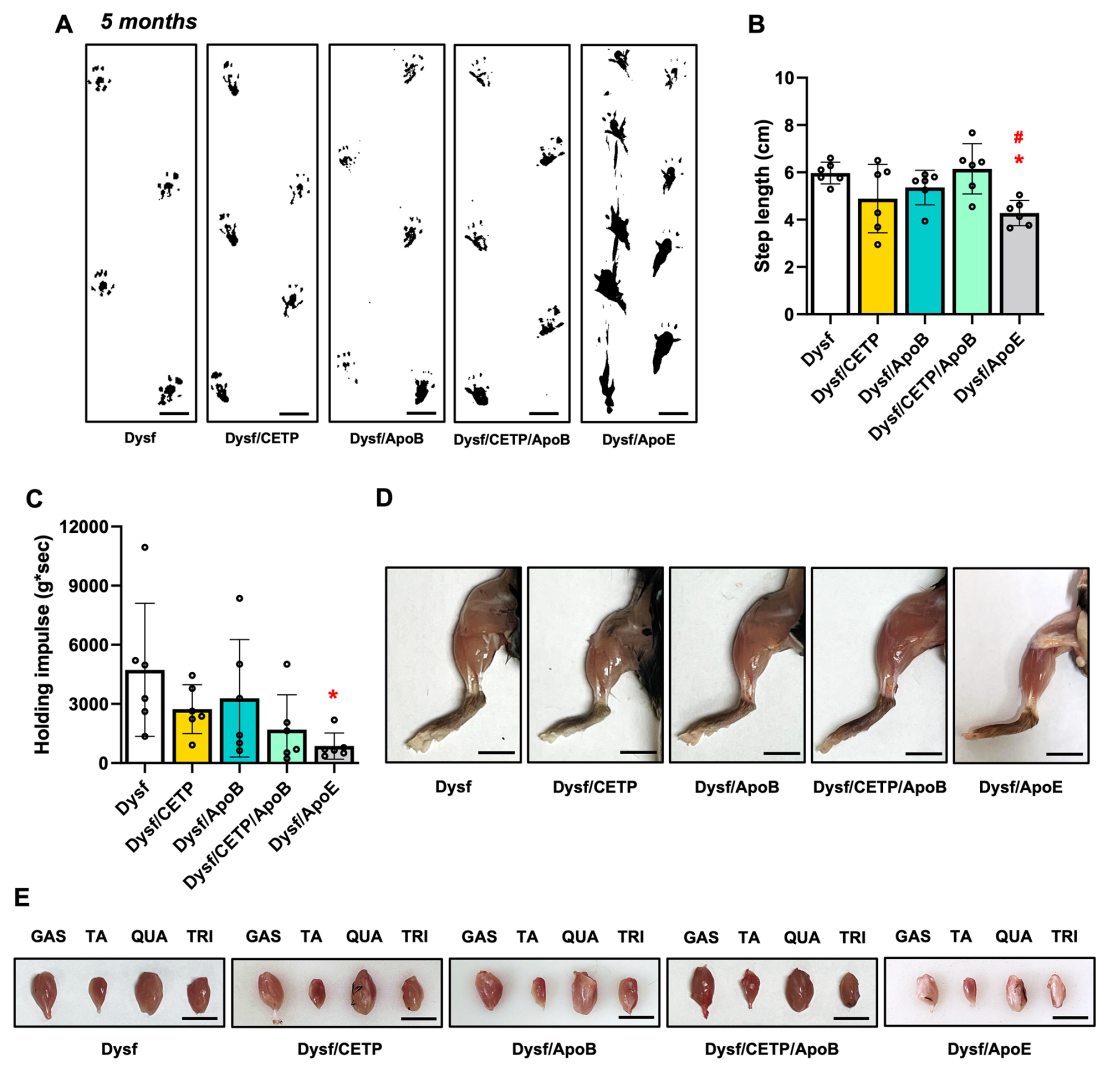
CETP/ApoB transgene did not affect muscle morphology or muscle function in 5-month-old adult Dysf mice. (**A**) Representative images of walking footprints of Dysf, Dysf/CETP, Dysf/ApoB, Dysf/CETP/ApoB, and Dysf/ApoE mice in gait tracking test. Scale: 1 cm. (**B**) Quantification of average step length (cm) in gait tracking test. (**C**) Quantification of holding impulse (g*sec) in hanging test. (**D**) Representative images of hindlimbs from Dysf, Dysf/CETP, Dysf/ApoB, Dysf/CETP/ApoB, and Dysf/ApoE mice. Scale: 1 cm. (**E**) Representative images of gastrocnemius (GAS), tibialis anteria (TA), Quadriceps (QUA), and triceps (TRI) muscles from Dysf, Dysf/CETP, Dysf/ApoB, Dysf/CETP/ApoB, and Dysf/ApoE mice. Scale: 1 cm. Data were presented as Mean ± SD. *P < 0.05 compared to Dysf; ^*#*^*P* < 0.05 compared to Dysf/CETP/ApoB

**Fig. 3:**
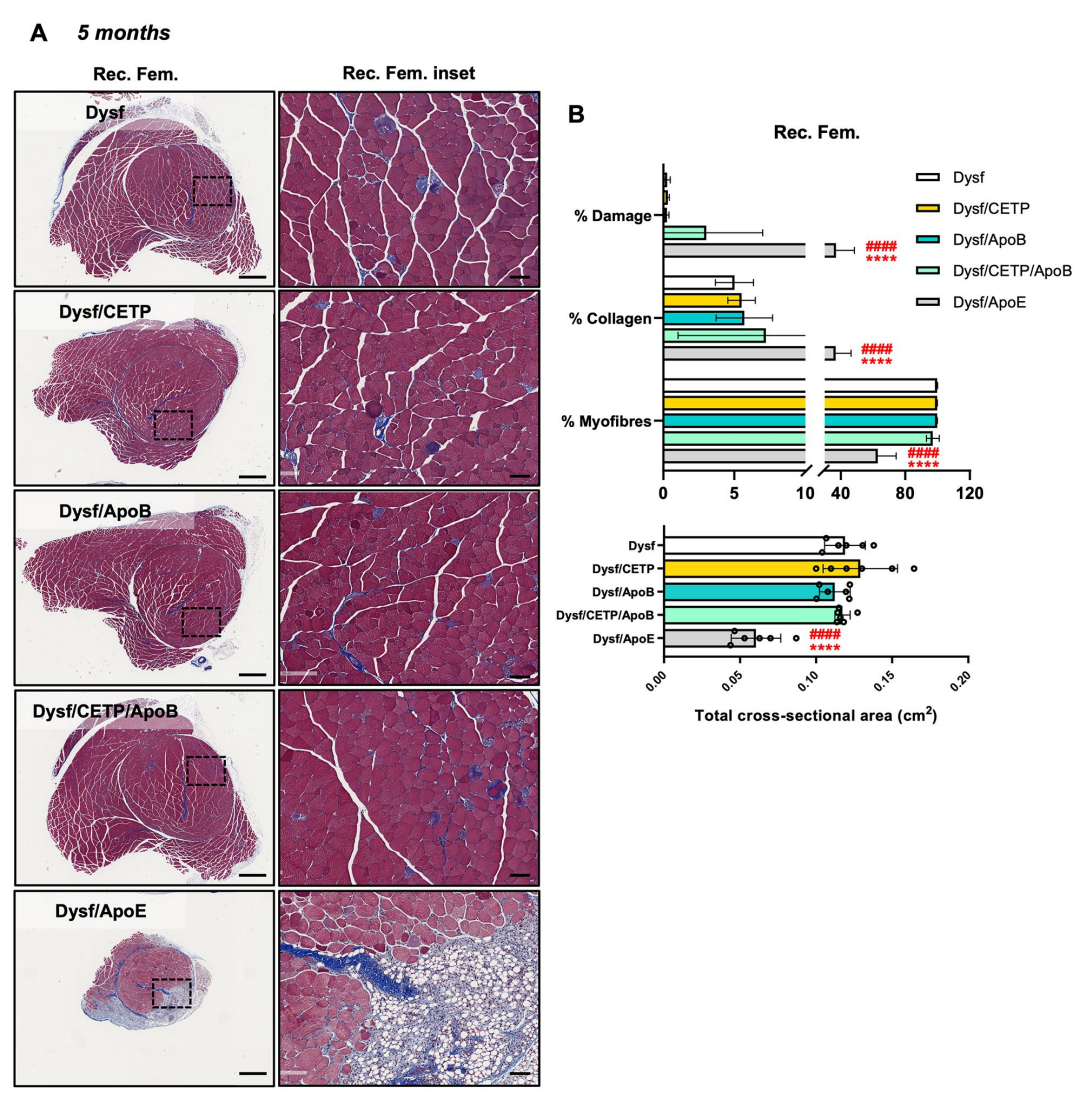
Expression of CETP/ApoB transgene lead to limited exacerbation of hindlimb muscle pathology compared to ApoE knockout in 5-month-old adult Dysf mice. (**A**) Representative images of Masson’s trichrome staining of rectus femoris (Rec. Fem.) muscles from Dysf, Dysf/CETP, Dysf/ApoB, Dysf/CETP/ApoB, and Dysf/ApoE mice and insets images. Scale: 1 mm for whole muscle images and 100 μm for insets. (**B**) Quantification of percentages of damaged area, collagen, healthy myofibres, and total area of Rec. Fem. muscles (*n* = 6). Data were presented as Mean ± SD. ****P < 0.0001 compared to Dysf; ^####^*P* < 0.0001 compared to Dysf/CETP/ApoB

**Fig. 4:**
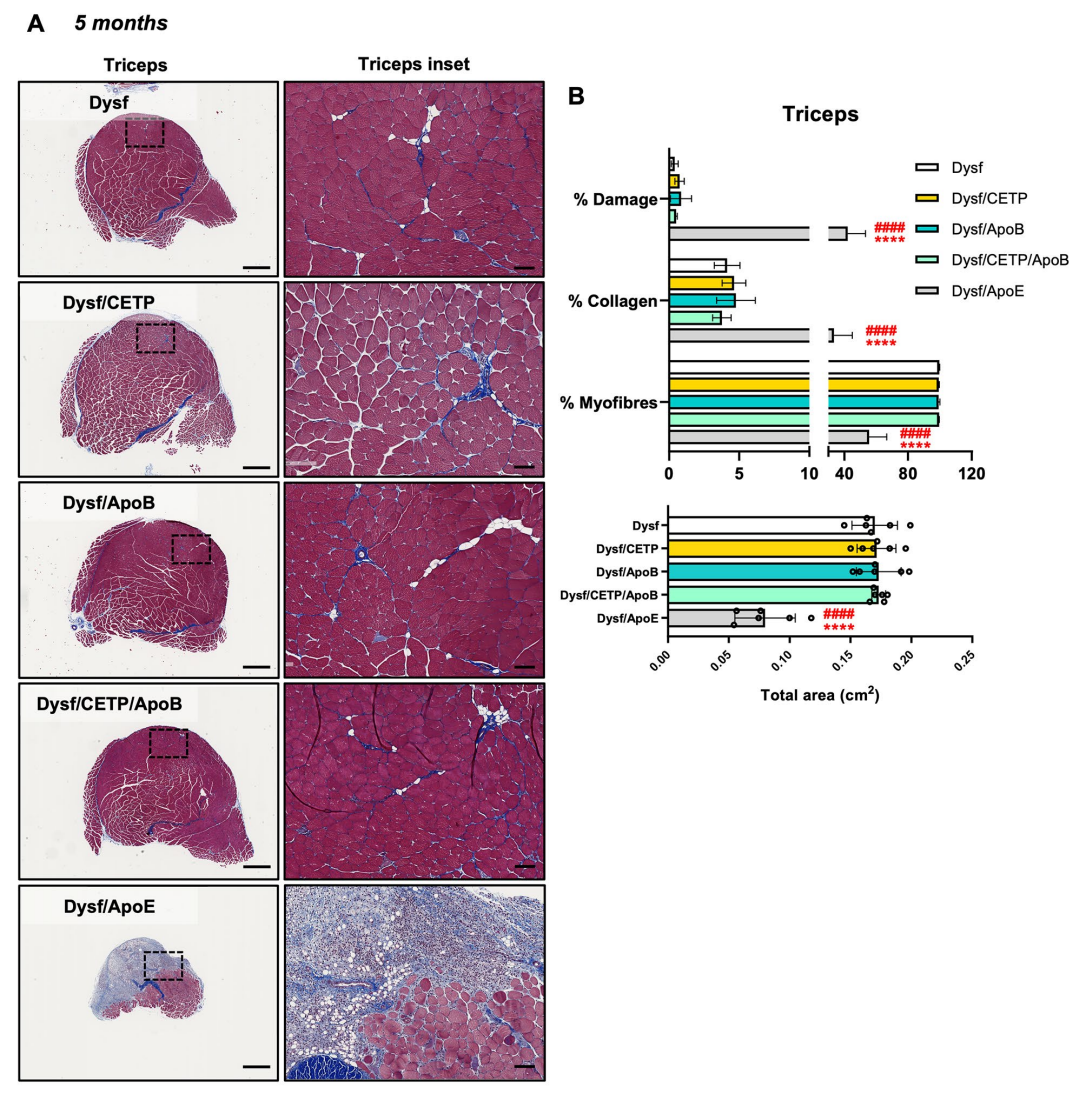
CETP/ApoB transgenes expression showed limited exacerbation of upper limb muscle pathology compared to ApoE knockout in 5-month-old adult Dysf mice. (**A**) Representative images of Masson’s trichrome staining of triceps muscles from Dysf, Dysf/CETP, Dysf/ApoB, Dysf/CETP/ApoB, and Dysf/ApoE mice and insets images. Scale: 1 mm for whole muscle images and 100 μm for insets. (**B**) Quantification of percentages of damaged area, collagen, healthy myofibres, and total area of triceps muscles (*n* = 6). Data were presented as Mean ± SD. ****P < 0.0001 compared to Dysf; ^####^*P* < 0.0001 compared to Dysf/CETP/ApoB

**Fig. 5:**
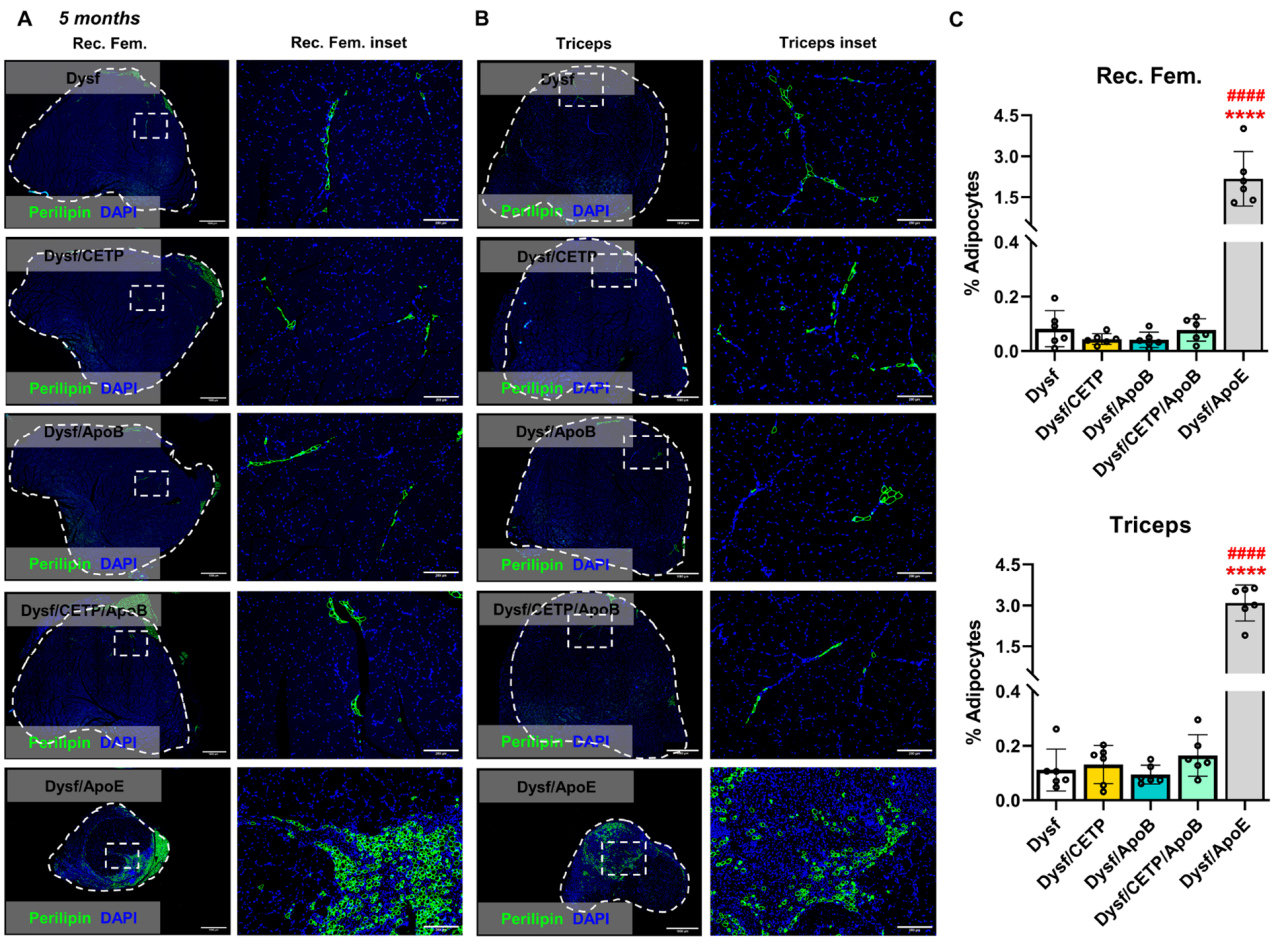
Perilipin staining of muscle sections from 5-month-old mice. (**A**) Representative images of rectus femoris (Rec. Fem.) from Dysf, Dysf/CETP, Dysf/ApoB, Dysf/CETP/ApoB, and Dysf/ApoE mice stained with Perilipin (Green) and insets images. (**B**) Representative images of triceps muscles. DAPI (blue) was used to visualize nucleus. Scale: 1 mm for whole muscle images and 200 μm for insets. (**C**) Quantification of percentages of adipocytes (perilipin positive area) in Rec. Fem. and triceps muscles (*n* = 6). Data were presented as Mean ± SD. ****P < 0.0001 compared to Dysf; ^####^*P* < 0.0001 compared to Dysf/CETP/ApoB

The role of CETP/ApoB expression on muscle homeostasis was assessed by characterizing ambulation, hanging capacity, and gross hindlimb morphology. As expected, 5-month-old Dysf/ApoE mice presented ambulatory dysfunction with a characteristic decrease in average step length compared to Dysf mice (5.97 ± 0.42 vs. 4.28 ± 0.49 cm, *P* < 0.05) and hindlimb dragging in severe cases, while CETP/ApoB expression did not cause stride length abnormalities (Fig. 2A-B). Dysf/ApoE mice also showed worse performance in hanging test compared to Dysf mice, while CETP/ApoB transgene did not cause significant hanging dysfunction (Fig. 2C). Following termination at 5 months, no difference was found between Dysf and Dysf/CETP/ApoB mice in body weight or muscle mass, while quadriceps and triceps from Dysf/ApoE mice showed significant mass reductions (Table 1). Compared to Dysf mice, Dysf/CETP, Dysf/ApoB, and Dysf/CETP/ApoB mice did not show obvious differences in general hindlimb and muscles (gastrocnemius (GAS), tibialis anterior (TA), quadriceps (QUA), triceps (TRI)) morphology. By contrast, age-matched Dysf/ApoE mice combined with a high cholesterol diet feeding showed severe muscle wasting, and muscles (GAS, QUA, TRI) were reduced in size and coated with fibro-fatty lesions (Fig. 2D-E). Masson’s trichrome staining revealed that compared to Dysf mice, CETP and ApoB-expressing mice did not show significant changes in total cross-sectional area and percentages of damaged area (blue and white), collagen (blue), and healthy myofibre area (red) of rectus femoris (Rec. Fem., inner part of QUA) (Fig. 3) and TRI muscles (Fig. 4) although CETP/ApoB-expressing mice presented limited exacerbation of Rec. Fem. damage (0.27% ± 0.20% vs. 3.03% ± 3.63%, *P* = 0.8875). By contrast, compared to Dysf or transgenic mice, Dysf/ApoE mice showed significant decreases in muscle cross-sectional area (49.2% and 48% of Rec. Fem., 53.1% and 54% of TRI, respectively) as well as increases of severe fatty-fibrotic infiltration (36.7% and 34.0% of Rec. Fem., 41.8% and 41.7% of TRI, respectively) (Figs. 3 and 4). Similar degree of muscle exacerbation among groups was further supported by H&E staining (Fig. S1). Moreover, perilipin staining showed that there was no difference in intramuscular adipocyte infiltration between Dysf and CETP/ApoB transgenic mice, while excessive lipid accumulation was seen in Dysf/ApoE mice (Fig. 5). These results suggest that transgenic CETP/ApoB expression in Dysf mice does not cause significant exacerbation, in stark contrast to Dysf/ApoE mice.

**Table 1:** Body weight and muscle mass of mice at 5 months

Group	Dysf	Dysf/CETP	Dysf/ApoB	Dysf/CETP/ApoB	Dysf/ApoE
Body weight (g)	28.29 ± 4.37	27.14 ± 3.79	30.47 ± 4.65	28.40 ± 3.55	25.27 ± 2.35
GAS (mg)	179.21 ± 31.02	170.04 ± 37.01	185.27 ± 37.61	180.92 ± 49.81	134.33 ± 22.09
TA (mg)	57.96 ± 15.07	58.98 ± 22.88	54.64 ± 11.17	48.92 ± 13.30	45.33 ± 11.91
QUA (mg)	233.54 ± 48.45	232.02 ± 60.03	254.60 ± 59.47	223.03 ± 68.83	92.00 ± 16.37^***##^
TRI (mg)	142.56 ± 25.60	146.96 ± 44.03	147.79 ± 34.14	143.13 ± 45.14	68.33 ± 17.04^**##^

Data were presented as mean ± SD. **P < 0.01, ***P < 0.001 compared to Dysf; ^##^*P* < 0.01 compared to Dysf/CETP/ApoB.

As CETP/ApoB transgene expression in Dysf mice did not cause major exacerbation of muscle pathology at 5 months of age, animals were aged until 11 months of age as previously described [9]. While reduced plasma HDL-C level and higher CHOL/HDL-C ratio were again presented in aged Dysf/CETP/ApoB mice, minor differences in plasma CHOL were observed between groups (Fig. S2). Meanwhile, muscle cross-sectional area, damage, collagen, myofibre area, and perilipin-labeled lipid accumulation of all CETP and/or ApoB-expressing experimental groups was not statistically different to that of Dysf control mice (Fig. S3, S4, S5, S6). These results indicated that persistently low level of plasma HDL-C via transgenic CETP/ApoB expression did not exacerbate muscle pathology in aged Dysf mice.

## Discussion

In contrast to most human MD that cause a decline in ambulation function before adulthood along with shorter life expectancy, rodent models of MD are generally afflicted by much milder phenotypes, with near-normal life span and mobility along with only mild tissue inflammation and fibro-fatty infiltration [22, 23]. Recent studies have suggested a causal relationship between MD severity and abnormalities in plasma lipoprotein and/or intramuscular cholesterol metabolism in human MD [8, 24–28]. Robust increases in CHOL and nonHDL-C through ApoE gene knockout lead to severe skeletal muscle wasting [13], providing not only critical insight into myofiber death in MD but also novel disease modeling options, although it must be noted that humans carry their high levels of nonHDL-C mostly in LDL particles despite expressing ApoE. In part due to the lack of CETP expression, mice have a much more HDL-C-rich lipid profile than humans [29, 30], which helps rationalize phenotype severity differences between human and rodent MD. Herein, human CETP and nonHDL-associated surface adapter protein human ApoB were expressed in mild Dysf mice. Despite reducing HDL-C similarly to LGMD2B, little to no exacerbation of the mild Dysf phenotype was observed. This suggests that in absence of high nonHDL-C levels, lowering of HDL-C through human CETP/ApoB expression is, from a modeling perspective of lesser value than expected.

Absence of significant MD exacerbation through human CETP/ApoB expression-mediated HDL-C lowering is an unexpected outcome of the current study, as HDL-C levels have been documented to affect muscle health. Higher HDL-C levels have been shown to be positively associated with elevated skeletal muscle mass in young males without changes in other parameters such as LDL-C and TG [31]. In another study on elder populations, increased HDL-C tertiles were found to correlate with better walking and physical function [32]. Notably, HDL-C level in Dysf/CETP/ApoB mice did not change with aging, which is consistent with human data [33]. However, human ApoB transgene tended to cause more muscle damage in aged Dysf mice, which was unexpectedly neutralized by CETP co-expression. The potential t response heterogeneity to human ApoB and CETP transgenes in LGMD2B might be of interest ase ApoB dyslipoproteinemia is also prevalent in hepatic steatosis, fibrosis, and fat infiltration [34–36]. On the other hand, data in this study are consistent with other results that showed CETP activity does not correlate to energy metabolism in liver or muscle [14], which indicates that the effect of CETP on tissue lipoprotein metabolism may be limited.

The severe muscle phenotype exhibited by Dysf/ApoE mice may also indicate that increased nonHDL-C is the critical factor to promote rapid progression of rodent MD. However, caution must be exercised when interpreting the lipid profiles of Dysf/ApoE mice from a clinical perspective. ApoE deficiency leads to robust, non-humanized increases in CHOL and nonHDL-C [13] that may also exacerbate a MD-specific intracellular cholesterol handling defect. In additions, although ApoE is predominantly expressed by the liver to regulate circulating lipids homeostasis, its expression in peripheral tissues such as adipocytes and macrophages [37, 38] may also interfere with whole-body lipid mobilization and inflammatory responses.

The circulating lipid profile humanization of rodent MD remains important from a modeling perspective. Other humanized rodent models of MD have been established through various genetic/dietary/environmental interventions [12, 21, 39], which partially mimicked muscle pathology and disease progression. In the current study, despite successfully lowering HDL-C level, the CETP/ApoB transgenes did not modify other lipid parameters such as CHOL or TG, which may be critical to successful MD severity expacerbation. t For instance, high TG/HDL-C ratio t is associated with more severe sarcopenia [40] and additional benefits from endurance exercise [41], suggesting the interaction between HDL-C and other lipoproteins may be of interest to to future studies that attempt to further refine rodent MD phenotypes. As intracellular cholesterol homeostasis is critical to myofiber integrity [42], approaches that humanize both circulating and intracellular cholesterol metabolism may be required to further improve MD severity. 

While transgenic CETP/ApoB expression in Dysf mice did not exacerbate muscle wasting, CETP/ApoB may nonetheless be part of future attempts to humanize rodent models of MD. As MD is increasingly recognized as a multi-faceted metabolic disease [43], improved modeling may also arise from combining circulating lipid-modulating approaches with other transgenic models that mimic MD abnormalities, such as telomerase RNA component (TERC) inactivation [44] or transforming growth factor-beta (TGF-β) overexpression [45]. As HDL-C is also critical to RCT by transporting excessive cholesterol from peripheral tissues such as skeletal muscles to the liver [46], a process that may be blunted in facioscapulohumeral MD [47], rodent MD phenotypes may be humanized by combining multiple lipid metabolism-altering approaches. Of note, patient gene polymorphism of key lipid metabolism regulators may also modulate disease progression akin to polygenic disease-modifying factors. For instance, CETP gene polymorphism can affect cardiovascular disease incidence [48, 49], which could also affect MD outcomes.

The option of treating MD patients with circulating lipid-modulating medications remains a source of controversy. Other studies have focused on treating MD using simvastatin, fenofibrate, catalpol, and ezetimibe [13, 50–52]. Statins can cause myopathies in about 10% of non-MD individuals as their main side-effect; however, they may help lower both nonHDL-C and free cholesterol accumulation in MD muscles [53]. In contrast, treating Dysf/ApoE and mdx/ApoE mice with ezetimibe provided evidence of the benefits and safety of specifically lowering nonHDL-C in LGMD2B and Duchenne MD [13]. HDL-C elevation therapies through CETP inhibition showed early potential in reducing cardiovascular events [54, 55], although most of the long-term trials have reported only minor improvements in CVD outcomes. Whether normalizing HDL-C or lowering CHOL and nonHDL-C in LGMD2B patients can delay loss of ambulation requires further investigation.

### Strengths and limitations

The current study is the first to explore the effect of lowering HDL-C via human CETP/ApoB transgenesis on muscle pathology in a rodent model of MD. Human CETP/ApoB transgenic Dysf mice were compared with Dysf/ApoE mice at two different time points(adult and old) to emphasize the influence of aging on the results. Lastly, muscle health was comprehensively evaluated by the combination of functional tests and multiple histopathological analyses with proper quantification.

The sample size in each group was limited due to the reduced fertility of the CETP/ApoB transgenic strain. Secondly, the absence of other analyses to detect subtle changes in muscle physiological activity and muscle/lipid metabolism-related gene/protein expression should be addressed in future studies. Moreover, this study was unable to rule out the possibility of changes in other lipid components due to genetic and dietary modifications. Thorough lipid profiling such as lipidomics should be considered.

## Conclusions

In conclusion, the current study demonstrated that HDL-C lowering via human CETP/ApoB expression does not lead to major muscle pathology or muscle function exacerbation in mild Dysf mice. In contrast, high CHOL or nonHDL-C levels caused by ApoE KO severely exacerbates muscle damage. These findings provide evidence that high CHOL, but not low HDL-C may be the culprit behind human LGMD2B disease severity, which is conducive to better guide the clinical monitoring and management of specific lipoprotein levels in LGMD2B patients. Future studies are needed to determine whether increasing HDL-C is of therapeutic values in LGMD2B patients and lowering HDL-C through CETP activity can further refine MD modeling in settings of high CHOL levels.

### Electronic supplementary material

Below is the link to the electronic supplementary material.

Supplementary Material 1

Supplementary Material 2

Supplementary Material 3

Supplementary Material 4

Supplementary Material 5

Supplementary Material 6

## Data Availability

The data used and/or analysed during the current study are available from the corresponding author on reasonable request.

## Abbreviations

MD: Muscular dystrophy
LGMD2B: Limb-girdle muscular dystrophy type 2B
CETP: Cholesteryl ester transfer protein
ApoB: Apolipoprotein B
ApoE: Apolipoprotein E
CHOL: Total cholesterol
HDL-C: High-density lipoprotein cholesterol
LDL-C: Low-density lipoprotein cholesterol
TG: Triglycerides
CVD: Cardiovascular disease
QUA: Quadriceps
Rec. Fem: Rectus Femoris
GAS: Gastrocnemius
TA: Tibialis anterior
TRI: Triceps
HMGCR: 3-hydroxy-3-methylglutaryl coenzyme A reductase
TGF-β: Transforming growth factor-beta
TERC: Telomerase RNA component
RCT: Reverse cholesterol transport
