# Supplementary figures and images for: Apolipoprotein E knockout, but not cholesteryl ester transfer protein (CETP)-associated high-density lipoprotein cholesterol (HDL-C) lowering, exacerbates muscle wasting in dysferlin-null mice
Source: Lipids Health Dis. 2024 Aug 13;23:247. doi: 10.1186/s12944-024-02227-5 (PMC11321019; doi:10.1186/s12944-024-02227-5)

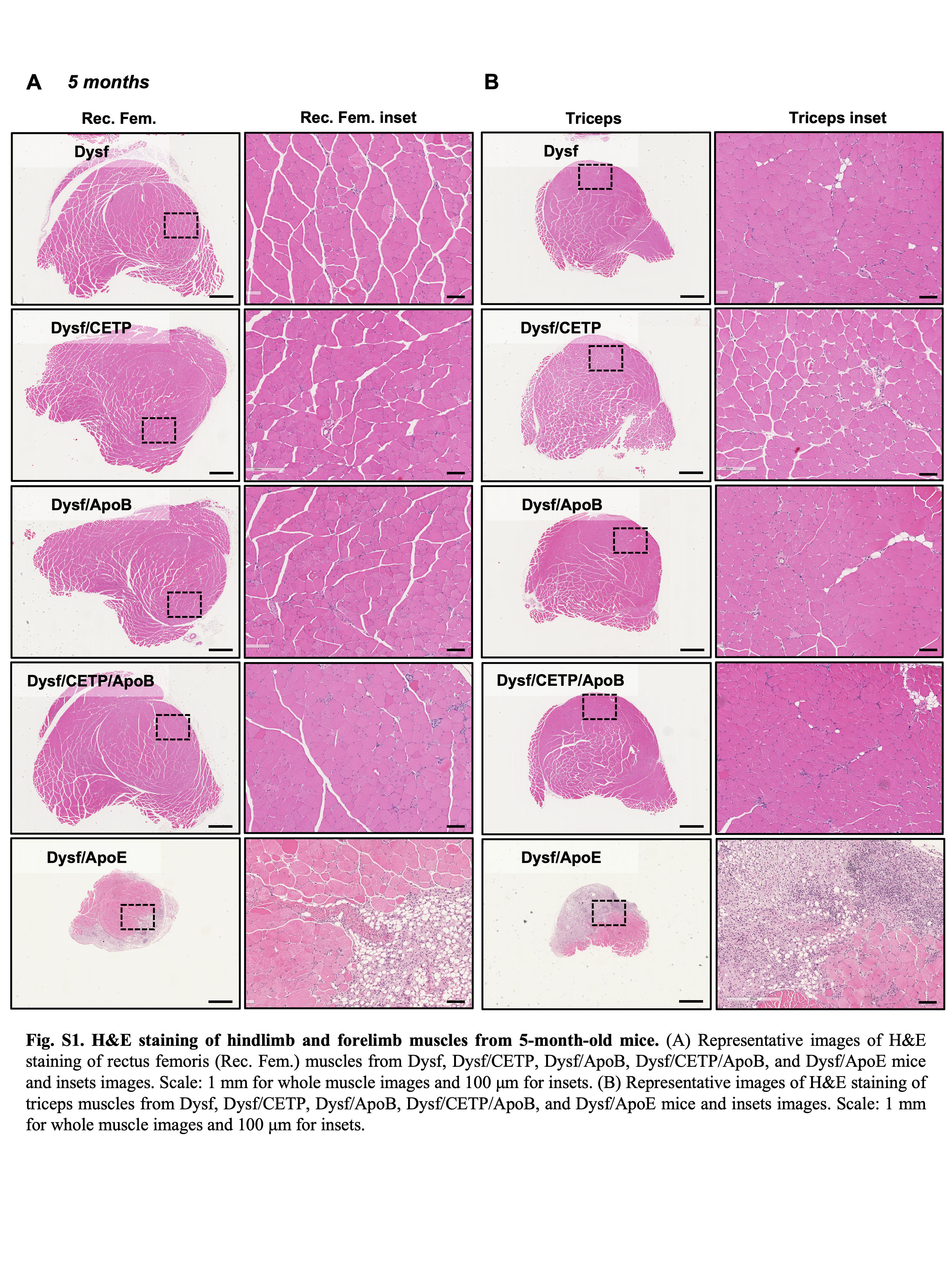

Supplement: Supplementary file 1 — Supplementary Material 1 [file 12944_2024_2227_MOESM1_ESM.png]

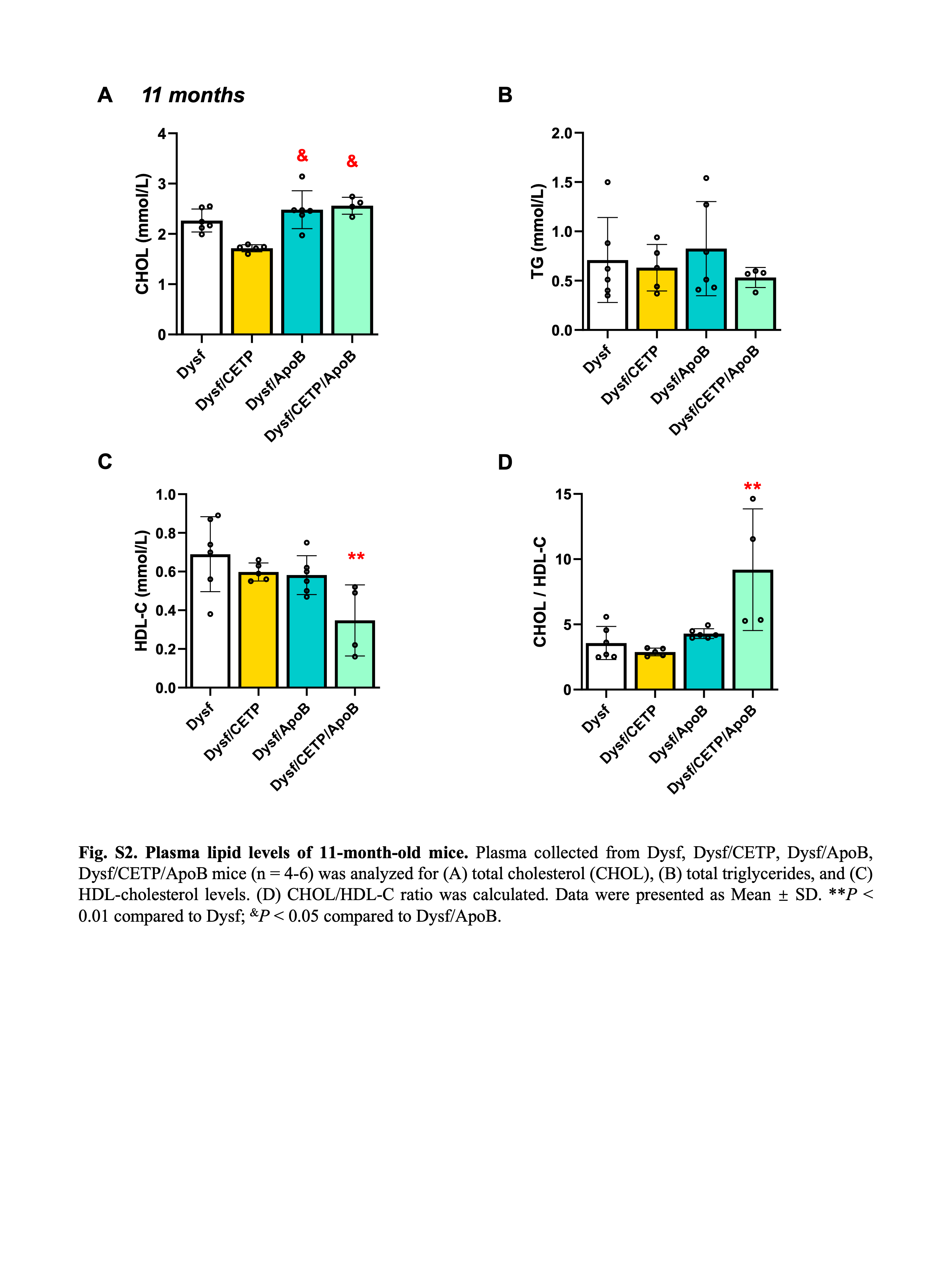

Supplement: Supplementary file 2 — Supplementary Material 2 [file 12944_2024_2227_MOESM2_ESM.png]

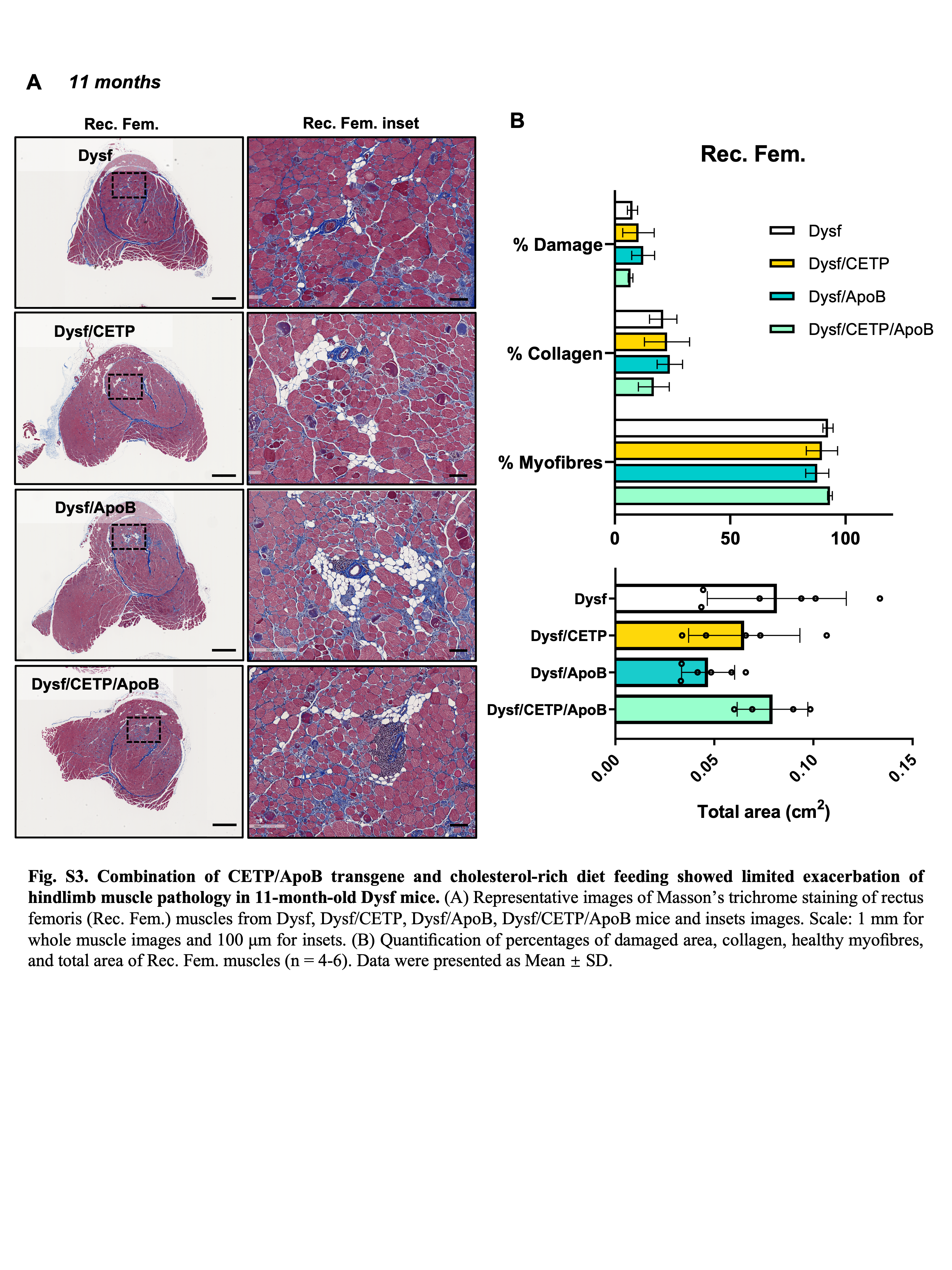

Supplement: Supplementary file 3 — Supplementary Material 3 [file 12944_2024_2227_MOESM3_ESM.png]

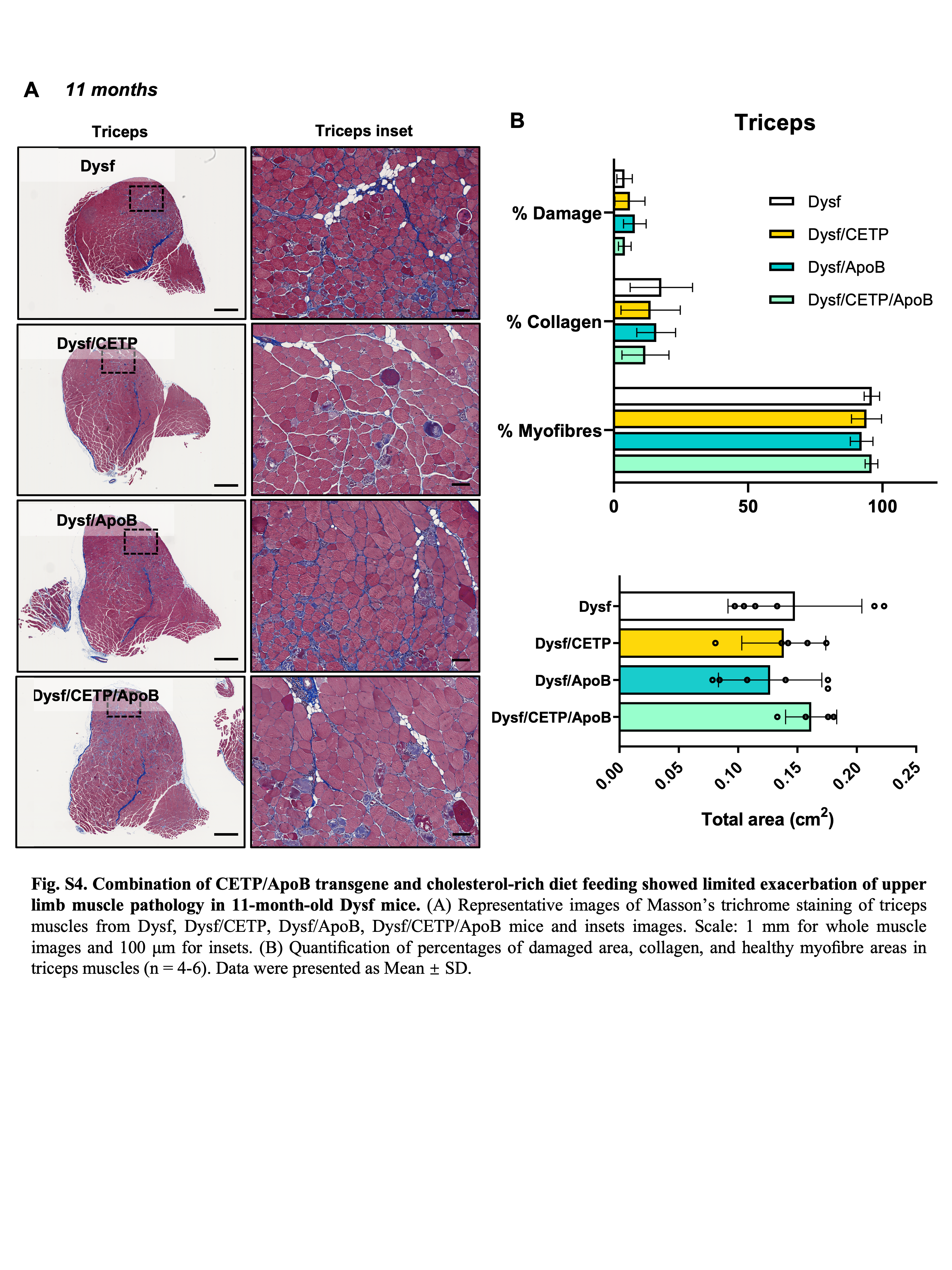

Supplement: Supplementary file 4 — Supplementary Material 4 [file 12944_2024_2227_MOESM4_ESM.png]

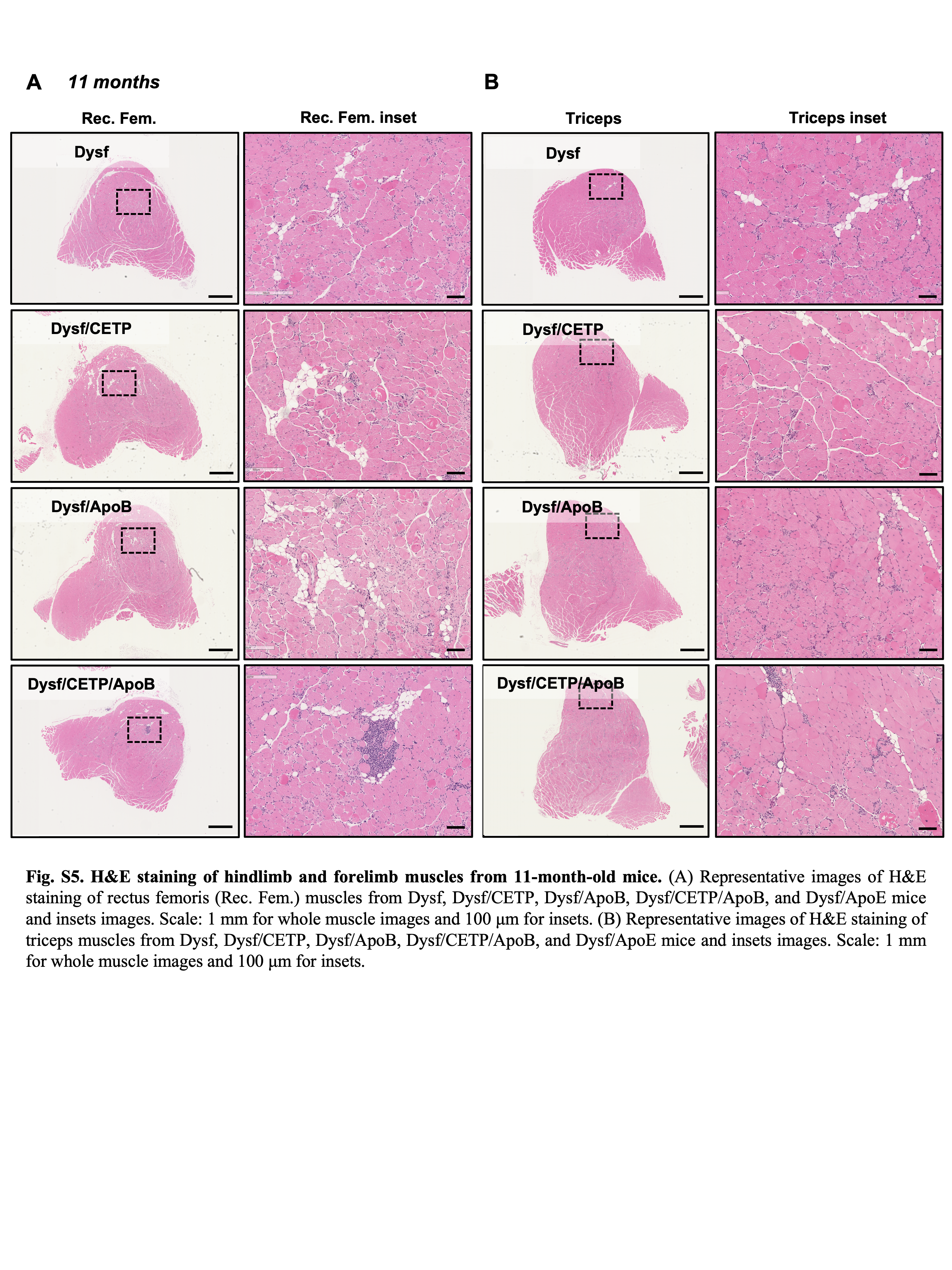

Supplement: Supplementary file 5 — Supplementary Material 5 [file 12944_2024_2227_MOESM5_ESM.png]

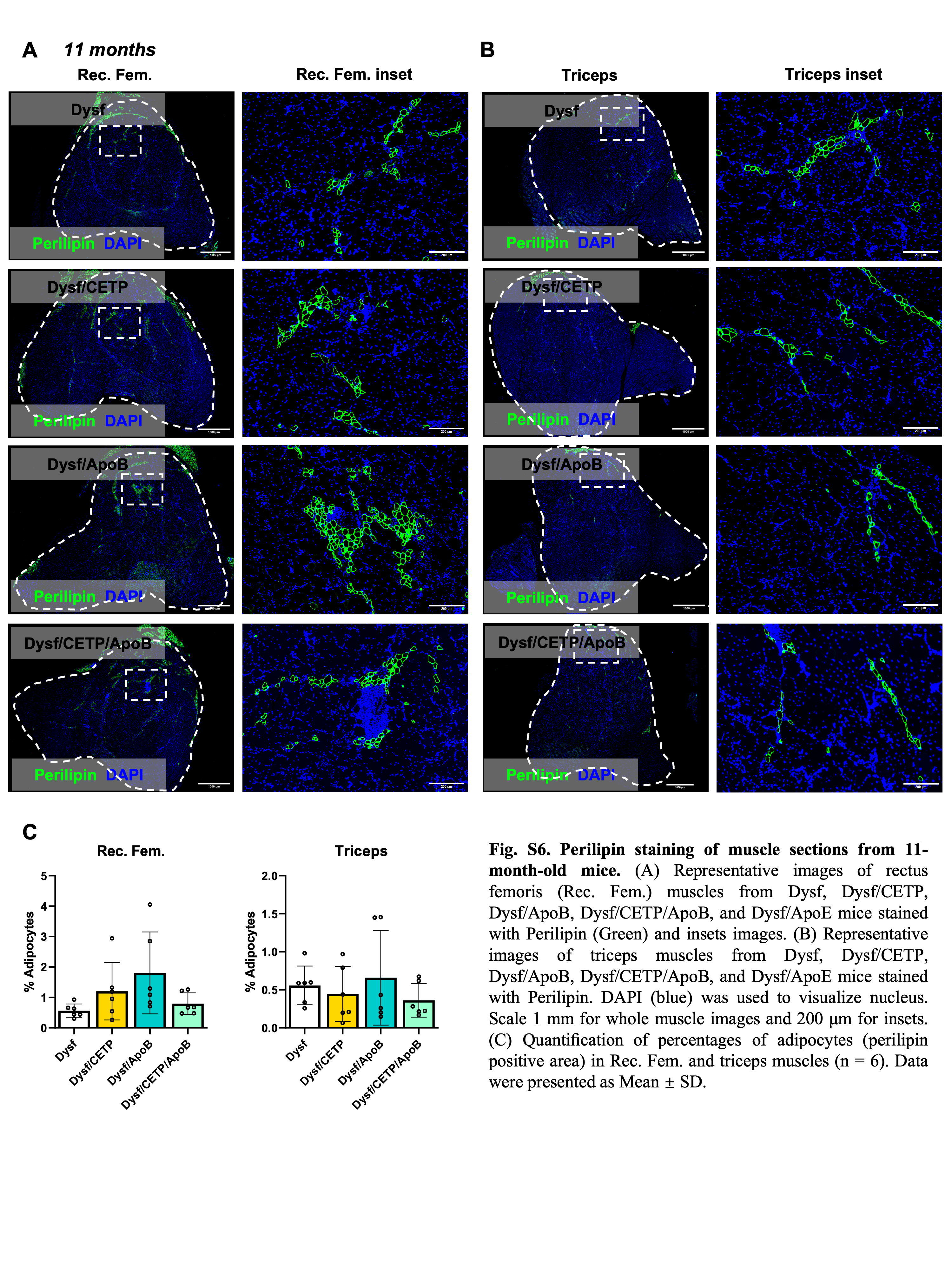

Supplement: Supplementary file 6 — Supplementary Material 6 [file 12944_2024_2227_MOESM6_ESM.png]
